# Linking academia and industry to bring drugs to the clinic: an interview with George Tidmarsh

**DOI:** 10.1242/dmm.012997

**Published:** 2013-07

## Abstract

George Tidmarsh, new Senior Editor on *Disease Models & Mechanisms* (DMM), is a physician with an academic science and medicine background and over two decades of industry experience at the cutting edge of biotechnology and drug development. Currently CEO of La Jolla Pharmaceutical Company Inc. as well as Consulting Associate Professor at Stanford University School of Medicine, he provides an insight into the value of industry as a conduit between academic research and patient benefit.

George Francis Tidmarsh was born in Oak Park, Illinois in 1960. He studied for an MD and PhD in Cancer Biology at Stanford University, where he also completed his clinical training in neonatology and pediatric oncology. He has over 20 years experience in the biotechnology industry and has been instrumental in guiding three drugs through the process of FDA approval. Currently CEO of La Jolla Pharmaceutical Inc., he is also Consulting Associate Professor of Pediatrics and Neonatology at Stanford University School of Medicine. During his career, George has served as CEO or President of several innovative biotechnology companies, including Threshold Pharmaceuticals Inc. and Horizon Pharma, Inc. (he founded both). During his time at Horizon, George invented and led the development of Duexis^®^, a drug approved by the FDA for the treatment of osteoarthritis and rheumatoid arthritis. In addition, George has been a senior officer within Coulter Pharmaceutical, Inc., where he led clinical development of the anti-cancer drug BEXXAR^®^, and SEQUUS Pharmaceuticals, Inc., where he led clinical development of Doxil^®^, another anti-cancer agent.

**Can you pinpoint one event or reason behind your decision to embark on a career in science?**

My interest in science dates back to my days as a seventh grader. My school had open classrooms and students were allowed to spend time in whatever subject they wanted. I found myself in science classes most of the time, drawn in by my natural fascination for the subject. The real turning point though was when I read *Biology of the Cancer Cell* by G. H. Heppner in high school. After that, I never gave another career a second thought.

**You have combined science, medicine and business very successfully. Did you have a plan early on in your career, or have you followed opportunities as they have arisen?**

My career path within science has been somewhat opportunistic with one thing following on from another. When I was applying to graduate school, my faculty sponsor advised that I should apply for the MD, PhD program. They offered to cover my tuition fees and to give me a stipend, so I accepted. Having embarked on the program, I was told that I, along with my peers, was responsible for ‘bridging the gap between the lab and the patient’, a message that I took very seriously at the time and have believed in ever since. When I was a pediatrics intern I decided I would like to spend some time back in the lab. More advice came, this time from my PhD advisor, who directed me to a company that he had started. Thus, I got my first taste of business.

**Figure:**
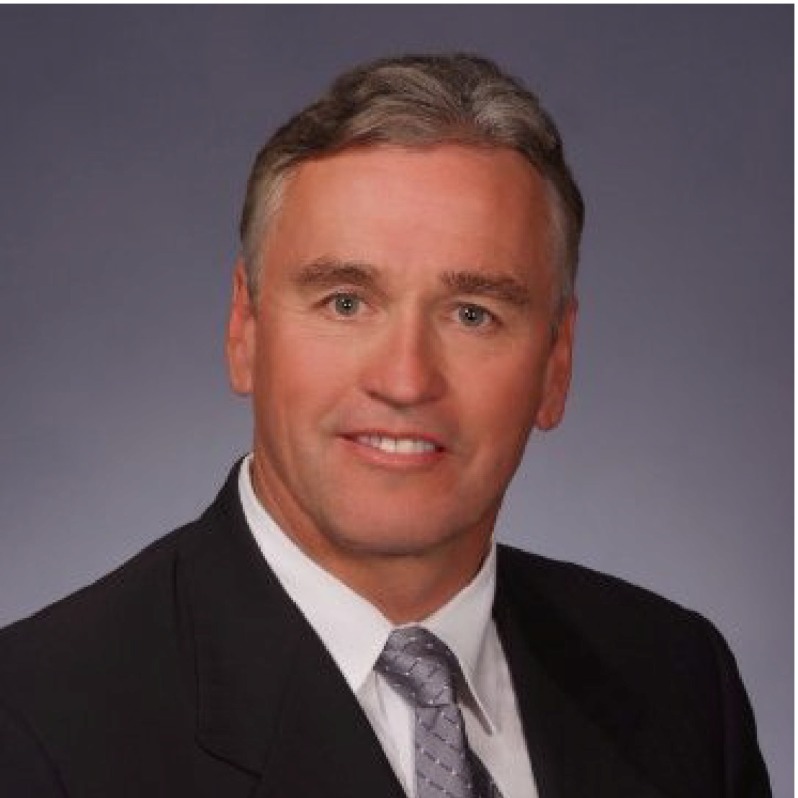


I’ve found that industry has provided a highly fertile ground in which to translate research findings into valuable clinical treatments, mostly due to the way science is funded. Clinical trials are expensive and most, particularly those for drug approval, are conducted in industry. By running clinical trials and managing the regulatory pathway that leads to new therapeutics, I have been able to bring new treatments to the clinic and fulfill my most important goal – to benefit patients.

**Which key people in science and in business have influenced you the most?**

Irv Weissman, my PhD advisor, for sure. He is a great scientist, and very creative. It was Irv who led me into the biotechnology industry. I would also say Bill Gates. I didn’t consider his relevance to me personally until I read a quote of his quite recently. He said: “Money has no utility to me beyond a certain point. Its utility is entirely in building an organization and getting the resources out to the poorest in the world”. That is definitely something I respect and aspire to.

**Thinking about your career to date, can you pick out the particular stepping stones that you consider as most significant in bringing you to where you are today?**

That moment when Irv Weissman directed me towards working in his biotech company is an obvious one. There have been many, many others, but perhaps a key decision was to come to Stanford. I seriously doubt I could hold the position I do within a university, combining that with research in industry, anywhere else in the country. In the late 1980s, the biotech industry was just burgeoning and Stanford provided the right environment for that to happen. My clinical fellowship at Stanford allowed me to look after patients to gain clinical experience but also to work in industry, at Gilead Sciences. There was a real open-mindedness here that made that possible. My current position at the university gives me a lot of satisfaction; I am able to use my industry contacts to get funding to help research from the university to generate the clinical data that will allow progression to the next stage. This is fairly rare position to be in.

**Do you think your position is unique?**

Not exactly unique, but certainly unusual. Other MDs working in the biotech industry are affiliated with a university, but most are not entrepreneurs – they haven’t founded their own companies using investment from venture capitalists. I hope that this will become less unusual in the future; in the current economic climate, getting research grants is a huge hurdle and young researchers face a difficult path. If I can play a small part in promoting and extending the positions available for clinicians who want to do research through industrial posts, it would be very satisfying.

**What is your life like at the moment – is there any such thing as a typical day for you?**

Currently, my work at La Jolla Pharmaceutical Inc. involves FDA interactions, clinical trial operations (working with investigators, developing protocols, enrolling patients), planning basic science experiments and talking with investors about our company. There is no typical day for me. One day I could be enrolling patients or running a clinical trial, while on another I could be involved in a presentation to current or prospective investors. These are very different activities and all require a very different approach, which makes life interesting, to say the least.

At Stanford, I work closely with the faculty members who trained me. In the neonatology division, I help find industry sponsorship for great scientific discoveries that need funding to get the next step toward approval. I recently identified a local biotechnology company that further developed a carbon-monoxide-sensing device pioneered at Stanford. In addition, I am working with a long-time mentor in the oncology department to help find a solution to the global shortage of oncology drugs.

“Data always means progress: if the data are positive, we can advance a new therapy to help patients. If the data are negative, we can then move in a new direction to develop and test a new hypothesis”

**What aspect of your work gives you the most satisfaction and why?**

Helping people whose lives are made difficult by illness is the main reason I do what I do. I can best achieve this goal by collecting and analyzing data and this gives me an enormous amount of satisfaction. Data always means progress: if the data are positive, we can advance a new therapy to help patients. If the data are negative, we can then move in a new direction to develop and test a new hypothesis. There is no such thing as a wrong answer; it’s about understanding what the results are telling us.

**What would you like to achieve in the next decade? What are your major goals?**

In the last 22 years I have played a large part in getting three new drugs through clinical trial to approval. I count those as among my greatest achievements to date and, in the coming years, I would like to bring further breakthrough therapies through that same approval process so that they can get to the people who need them. This is by far the most important goal for me.

Also, I would like to help find a solution to the global oncology drug shortage. In some cases, cancer patients are being forced to deal with treatment changes, delays and life-threatening complications due to the lack of access to inexpensive, generic medicines. The surprising part about this is that the problem exists not only in less-developed countries but also in the United States.

**How did you become involved with DMM and what made you accept the role as one of the Senior Editors?**

Ross [Cagan, Editor-in-Chief at DMM] was referred to me by a colleague who was involved with one of the companies that I founded. I accepted the role to help bring scientific credibility to the work that scientists perform in industry. When I was a graduate student, the prevailing opinion was that scientists who chose industry as a career were in some way second rate but I have never agreed that this is the case. I believe that a great deal of brilliant science takes place in industry and I think that needs to be better appreciated by the scientific community as a whole.

“Animal models of diseases are absolutely critical in the drug development process... Bad models are bound to lead to bad decisions, so the better models we have, the more likely we are to discover and cost-effectively develop drugs that work well to help patients”

**What do you feel is the main benefit of the journal in this field of science and how would you like to contribute in your new role?**

Animal models of diseases are absolutely critical in the drug development process. Data collected in these models can help determine if we should test the therapy in humans. This is a critical step for the well-being of the patients and the economy of the process as a whole. The step from animals to humans is not a cheap one – that decision costs $10–15 million at least. It needs to be made after careful consideration of all the available information, and data from disease models contribute significantly. Bad models are bound to lead to bad decisions, so the better models we have, the more likely we are to discover and cost-effectively develop drugs that work well to help patients.

**How do you relax and have fun away from the lab and the boardroom?**

My family is my number one way to relax. I play ultimate frisbee or football with my three boys as many evenings as possible. I am also a competitive long-distance swimmer.

**If you had to give up what you do tomorrow and switch career completely, what would you be most likely to do?**

I can think of nothing more rewarding than being a doctor on the front lines, taking care of patients day-to-day.

**What one thing would people be surprised to learn about you?**

That I whole-heartedly agree with Bill Gates’ philosophy: money is not important to me beyond having enough to live simply and take care of my family. It is a means to help other people and that is all.

